# Enhancing Oncolytic Adenovirus Replication by Early Region 1A Protein‐Mediated Degradation of E1A Binding Protein p300

**DOI:** 10.1002/mco2.70683

**Published:** 2026-03-18

**Authors:** Boduan Xiao, Qingzhe Yang, Shichuan Hu, Jianchuan Hu, Zhongbing Qi, Yao Zhang, Yu Qin, Ping Cheng

**Affiliations:** ^1^ Department of Biotherapy，Cancer Center and State Key Laboratory of Biotherapy West China Hospital Sichuan University Chengdu People's Republic of China

**Keywords:** E1A binding protein p300, early region 1A protein, oncolytic adenovirus, viral replication

## Abstract

Oncolytic adenovirus (OAd) therapy is one of the effective treatment strategies for solid malignant tumors, and E1A is a requirement for adenovirus replication. Thus, it is very important to study how E1A regulates adenovirus replication. The p300 and E1A expression were detected by Western blot. The viral replication of OAd was detected by virus replication assay. The interaction between E1A and p300 was analyzed by immunofluorescence and immunoprecipitation assays. The therapeutic effect of OAd‐shp300 was analyzed by MTT assay and animal experiments. The results indicated that OAd infection or E1A overexpression could reduce p300 expression, implying that OAd might reduce p300 expression via E1A, and p300 knockdown could enhance viral replication and cell cytotoxicity of OAd. Furthermore, E1A promoted viral replication of OAd via mediating p300 ubiquitination degradation to inhibit the IFI16/STING/IRF3/IFN‐β signaling pathway. Additionally, OAd‐shp300 induced highly efficient viral replication and potent antitumor activity both in vitro and in vivo. In this study, OAd can reduce p300 expression by promoting its ubiquitination via E1A, thereby enhancing viral replication and cell cytotoxicity. Therefore, this study can provide a biomarker for screening patients who are sensitive to OAd and new ideas for clinical tumor treatment.

## Graphical abstract

E1A is an important protein for adenovirus to initiate viral replication. Previous reports have shown that E1A binds to p300 as a regulatory factor. However, our study provides a new perspective that E1A promotes oncolytic adenovirus replication by degrading p300. Therefore, the findings of this study are of great significance for understanding adenovirus replication. We have also developed a highly replicable oncolytic adenovirus based on this study to treat tumors with high p300 expression. In clinics, p300 as a biomarker is used to screen for patients who are more likely to respond well to oncolytic adenovirus treatment.

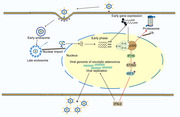

## 1 Introduction

Oncolytic virus therapy is among the most promising treatment strategies for solid malignant tumors [1, 2]. Oncolytic viruses include oncolytic adenovirus (OAd) [3, 4], oncolytic vaccinia virus [5, 6], and oncolytic herpes simplex virus [7, 8]. These oncolytic viruses have been observed to specifically proliferate in tumor cells and cause lysis. Among these oncolytic viruses, OAd has been used in the clinical treatment of tumors and has achieved better therapeutic effects [9]. However, some patients remain resistant to the OAd therapy. Therefore, investigating the biology of OAd is essential to distinguish between OAd‐sensitive and non‐sensitive patients.

The OAd replication is the basis of oncolysis, and early region 1A protein (E1A) is necessary for adenovirus replication [10]. However, E1A alters the gene expression of host cells or regulates viral gene expression, enabling the virus to replicate [11, 12]. For instance, E1A of adenovirus targets the DREF nuclear factor to modulate virus gene expression, growth, and replication [13]. Furthermore, it primarily functions by interacting with various host factors [14]. Therefore, evaluating the E1A interaction with new host factors is essential for identifying novel viral replication modulatory mechanisms.

E1A binding protein p300 (p300) is a histone acetyltransferase that catalyzes the acetylation of histones and various non‐histone proteins, promotes chromosome structure loosening, and initiates transcription. Furthermore, it acts as a transcriptional activator and has been found to interact with adenovirus E1A [15]. Moreover, it has been revealed that adenovirus functions by associating E1A with p300. For instance, small E1A of adenovirus uses tumor suppressor Rb and lysine acetylases p300/CBP to inhibit target host genes and promote viral infection [16]. Adenovirus E1A conservative Region 3 regulates transcription through p300/CBP [15]. In addition, E1A binding with p300 has been found to effectively promote adenovirus replication [17]. However, how E1A binds p300 to promote adenovirus replication remains elusive.

Previous studies have shown that E1A binds to p300 as a transcription factor to regulate gene expression in host cells [16] or OAd [15], promoting virus replication. However, this study revealed that in cells with low p300 expression, OAd's replication ability is enhanced, and p300 expression is degradated decreased in OAd‐infected cells. Additionally, E1A promotes adenovirus viral replication by binding to and degradating p300, suggesting E1A may not play the role of a transcription factor. Therefore, this study showed that p300 might be a factor limiting the OAd replication and act as a biomarker for screening patients who are sensitive to OAd.

## 2 Result

### 2.1 Oncolytic Adenovirus Exhibits Stronger Viral Replication and Cell Cytotoxicity in Low p300 Expression Cells

**FIGURE 1:**
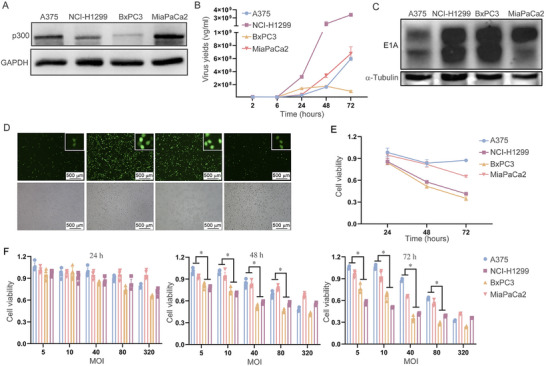
OAd replication and cytotoxicity are enhanced in cells expressing low levels of p300. (A) p300 levels were detected by Western immunoblotting. (B) qPCR was used to analyze viral replication. (C) Following OAd treatment for 24 h, E1A levels were detected via Western immunoblotting. (D) Following OAd‐EGFP treatment for 24 h, cells were imaged via fluorescence microscopy. (E, F) An MTT assay was used to quantify viability. *p < 0.05. GAPDH and α‐tubulin served as the internal control.

Previous studies have indicated that the binding of E1A to p300 contributes to the replication of adenovirus [17]. Furthermore, E1A has been found to bind p300 as a transcription factor [15]. Therefore, to investigate the association of p300 and OAd replication, the expression of p300 was detected in different cells. The result showed that p300 expression is highest in MiaPaCa2 cells and lowest in BxPC3 cells (Figure 1A). From Figure 1B, it can be seen that the viral replication of OAd first reached the endpoint of viral replication in BxPC3 cells, that is, after 24 h, there is no change in viral replication.Correspondingly, at 24 h, OAd replication in MiaPaCa2 and A375 cells lagged behind that in BxPC3 and NCI‐H1299 cells, suggesting that OAd has a stronger replication ability in low p300 expression cells (Figure 1B). E1A expression was also increased in the lower p300 expression cells (NCI‐1299 and BxPC3) compared to higher p300 expression cells (A375 and MiaPaCa2) at 24 h post‐OAd infection (Figure 1C). Immunofluorescent imaging further revealed stronger EGFP fluorescence in the lower p300 expression cells (NCI‐1299 and BxPC3) compared to higher p300 expression cells (A375 and MiaPaCa2) (Figure 1D). Moreover, compared to high p300 expression MiaPaCa2 and A375 cells, OAd showed stronger cytotoxicity in low p300 expression BxPC3 and H1299 cells (Figure 1E,F). Overall, OAd exhibits stronger viral replication and cell cytotoxicity in cells with low p300 expression.

### 2.2 The Expression of p300 Was Degradated by E1A‐Mediated Ubiquitination

**FIGURE 2:**
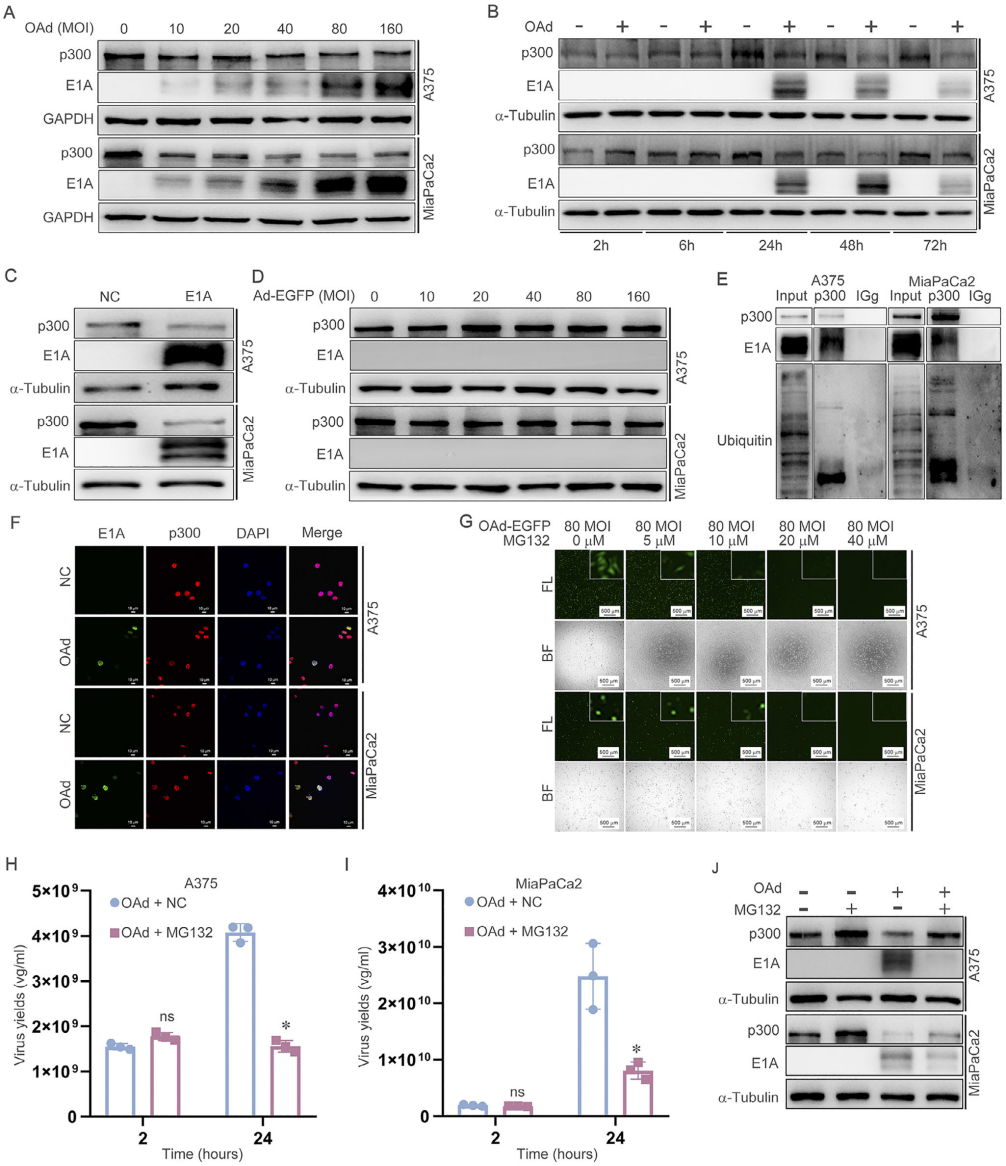
The expression of p300 was degradated by E1A‐mediated ubiquitination. (A) Following OAd treatment at different MOIs, p300 and E1A levels were detected via Western immunoblotting. (B) Following OAd treatment for different amounts of time, p300 and E1A levels were detected via Western immunoblotting. (C) p300 and E1A levels were detected via Western immunoblotting in cells overexpressing E1A. (D) Following non‐replicating adenovirus (Ad) treatment, p300 and E1A levels were detected via Western immunoblotting. (E) An immunoprecipitation approach was used to detect p300 binding to E1A and p300 ubiquitination. (F) Immunofluorescence was used to detect the binding of p300 to E1A. (G) Following OAd‐EGFP and MG132 treatment for 24 h, cells were imaged via fluorescence microscopy. (H, I) qPCR was used to detect viral replication in A375 and MiaPaCa2 cells with OAd and MG132 treatment at 2 and 24 h. (J) Following OAd‐EGFP and MG132 treatment for 24 h, p300 and E1A levels were detected via Western immunoblotting. *p < 0.05. GAPDH and α‐tubulin served as the internal control.

Based on the above results, it was hypothesized that OAd affects its replication by affecting p300 expression. To validate this hypothesis, MiaPaCa2 and A375 cells were infected with OAd (MOI = 10, 20, 40, 80, and 160), and p300 expression was measured at 2, 6, 24, 48, and 72 h. The data indicated that p300 expression decreased as MOI or time increased (Figure 2A,B). Moreover, E1A overexpression was associated with decreased p300 expression (Figure 2C), and no change was observed in p300 expression after infection with non‐replicating adenovirus of different MOIs (Figure 2D), suggesting that E1A can reduce p300 expression. To investigate how E1A reduces p300 expression, immunofluorescence and immunoprecipitation analyses were conducted, which revealed that E1A could bind p300 (Figure 2E,F), which was consistent with earlier studies [15]. Furthermore, ubiquitin was detectable when lysates were precipitated with anti‐p300 (Figure 2E). To determine whether E1A is capable of decreasing p300 expression through a ubiquitination‐related process, cells were treated with the proteasome inhibitor MG132. Increased MG132 concentrations coincided with a drop in EGFP fluorescence intensity, suggesting that ubiquitination may hamper viral replication (Figure 2G). MG132 (20 µM) treatment also reduced degradation of p300 in infected cells, suggesting that E1A can reduce p300 expression through the ubiquitination of p300 (Figure 2H,I). MG132 treatment at this same dose also suppressed OAd replication, further supporting the ability of OAd infection to promote viral replication through the E1A‐mediated ubiquitination of p300 (Figure 2J).

### 2.3 p300 Knockdown Can Enhance Viral Replication and Cell Cytotoxicity of Oncolytic Adenovirus

**FIGURE 3:**
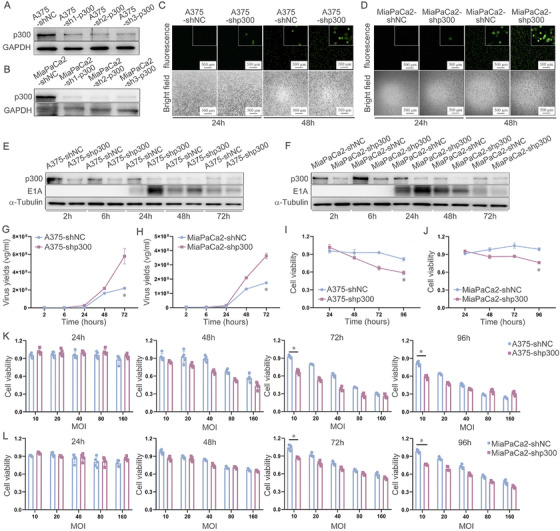
p300 knockdown can enhance replication and cell cytotoxicity of oncolytic adenovirus. (A, B) Western immunoblotting was used to detect p300 in cells following its knockdown. (C, D) Following OAd‐EGFP, fluorescence microscopy imaging was performed after 24 and 48 h. (E, F) Following OAd treatment for the indicated times, Western immunoblotting was used to detect p300 and E1A in cells in which p300 had been knocked down. (G, H) Following OAd treatment for the indicated periods, qPCR was used to detect viral replication in p300 knockdown cells. (I, J) Following OAd treatment for the indicated periods, an MTT assay was used to assess the viability of p300 knockdown cells. (K, L) Following OAd treatment at various MOIs, an MTT assay was used to assess p300 knockdown cell viability. *p < 0.05. GAPDH and α‐tubulin served as the internal control.

To further verify whether OAd's replication ability is related to low p300 expression, p300 was knocked down in cell lines. Figure 3A,B shows the successful establishment of the p300 knockdown cell lines A375‐sh1‐p300 (A375‐shp300) and MiaPaCa2‐sh1‐p300 (MiaPaCa2‐shp300), which were used for subsequent experiments. Immunofluorescent imaging revealed stronger EGFP fluorescence in the A375‐shp300 and MiaPaCa2‐shp300 cells in which p300 was knocked down, relative to corresponding control cells that exhibited stronger fluorescence intensity compared with the control cells (A375‐shNC and MiaPaCa2‐shNC) at 24 and 48 h following OAd‐EGFP infection (MOI = 20) (Figure 3C,D). The silencing of p300 is thus an effective means of enhancing OAd viral replication. E1A expression was also increased in these p300‐knockdown cells at 24 h post‐OAd‐EGFP infection (MOI = 20) (Figure 3E,F). Notably, compared to the control group, the expression of E1A was increased in the p300 knockdown group (A375‐shp300 and MiaPaCa2‐shp300) at 24 h after infection with OAd‐EGFP (20 MOI), and E1A subsequently started to decrease, speculating that high cytopathy leaded to cell lysis and release of intracellular contents into the supernatant (Figure 3E,F). Viral replication was further confirmed to be enhanced in these cells exhibiting low levels of p300 expression (Figure 3G,H). Altogether, it was inferred that p300 knockdown could enhance OAd replication. Additionally, to validate that low p300 expression cells have stronger toxicity of OAd, an MTT assay was conducted. The data revealed reduced cell viability in the p300 knockdown group (A375‐shp300 and MiaPaCa2‐shp300) compared with the control group (A375‐shNC and MiaPaCa2‐shNC) at 24, 48, 72, and 96 h post‐infection with OAd (MOI = 10) (Figure 3I,J). OAd infection with different MOI revealed that low MOI had stronger cytotoxicity in the low p300 expression group (Figure 3K,L). Therefore, it was inferred that p300 knockdown can enhance the cytotoxicity of OAd.

### 2.4 High p300 Expression Exhibits Negative Regulation of Viral Genome Replication

**FIGURE 4:**
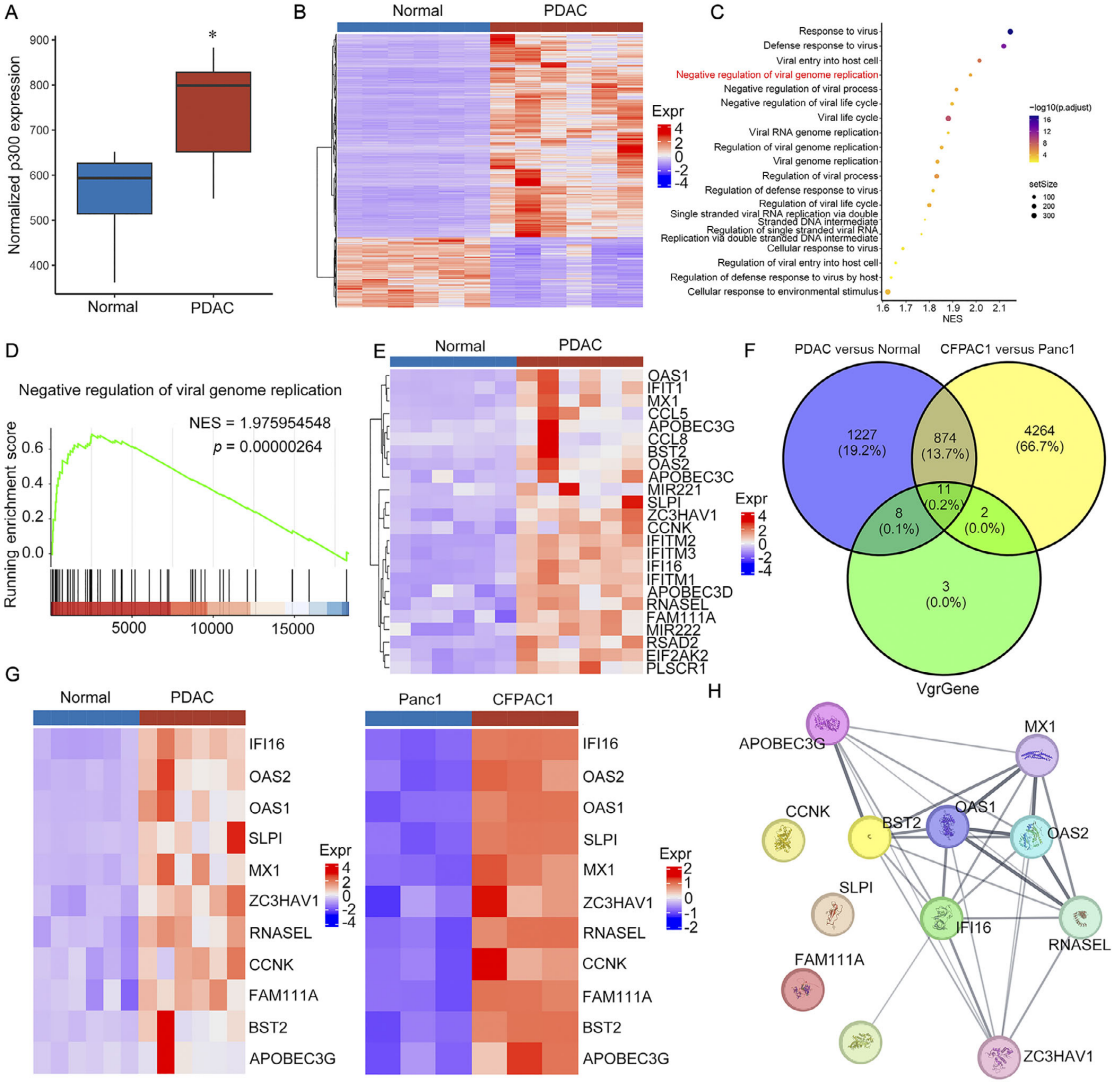
High p300 expression exhibits negative regulation of viral genome replication. (A) p300 levels were upregulated in PDAC. (B) Heatmap of DEGs between PDAC tissue samples and normal pancreatic tissue samples. (C) GSEA biological process analysis was used to analyze virus‐related biological processes. (D) GSEA of the negative regulation of viral genome replication in PDAC patients with high p300 expression. (E) Heatmap of DEGs in negative regulation of viral genome replication. (F) The Venn diagram of intersection analysis of DEGs about negative regulation of viral genome replication in pancreatic cancer tissue data (GSE41372) and pancreatic cancer cell data (GSE165949). (G) Heatmap of DEGs about negative regulation of viral genome replication in the Venn diagram of intersection analysis. (H) The relationship between p300 and genes related to negative regulation of viral genome replication was analyzed by STRING website.

To explore whether high p300 expression is related to antiviral ability, we analyzed patient samples (GSE41372) of pancreatic ductal adenocarcinoma (PDAC) through bioinformatics analysis. p300 was overexpressed in the PDAC tissue samples compared with normal pancreatic tissue samples (Figure 4A). Heatmap showed differentially expressed genes (DEGs) between PDAC tissue samples and normal pancreatic tissue samples (Figure 4B). Further, gene set enrichment analysis (GSEA) biological process analysis showed that PDAC tissue samples had negative regulation of viral genome replication (Figure 4C,D). Moreover, heatmap (Figure 4E) and the volcano plot (Figure S1) showed DEGs in negative regulation of viral genome replication. To screen for more accurate DEGs in negative regulation of viral genome replication, the higher p300 expression cells (CFPAC1) and lower p300 expression cells (Panc1) were applied through bioinformatics analysis (Figure S2A). Notably, compared to CFPAC1 cells, OAd had stronger viral replication ability in Panc1 cells (Figure S2B). The results of bioinformatics analysis showed that p300 was overexpressed in the CFPAC1 cells compared with Panc1 cells (Figure S3). The Venn diagram of intersection analysis of pancreatic cancer tissue data (GSE41372) and pancreatic cancer cell data (GSE165949) showed that there were 11 common DEGs about negative regulation of viral genome replication, accounting for 0.2% of the total genes (Figure 4F). Moreover, heatmap showed 11 common DEGs in negative regulation of viral genome replication in the pancreatic cancer tissue and cells (Figure 4G). STRING website (https://cn.string‐db.org/) was used to analyze the relationship between p300 and genes related to negative regulation of viral genome replication (Figure 4H), indicating that p300 mainly affected IFI16, thereby affecting genes related to negative regulation of viral genome replication. Studies have shown that IFI16 controls virus replication [18].

### 2.5 p300 Regulates Viral Replication of Oncolytic Adenovirus by Modulating the IFI16/STING/IRF3/IFN‐β Signaling Pathway

**FIGURE 5:**
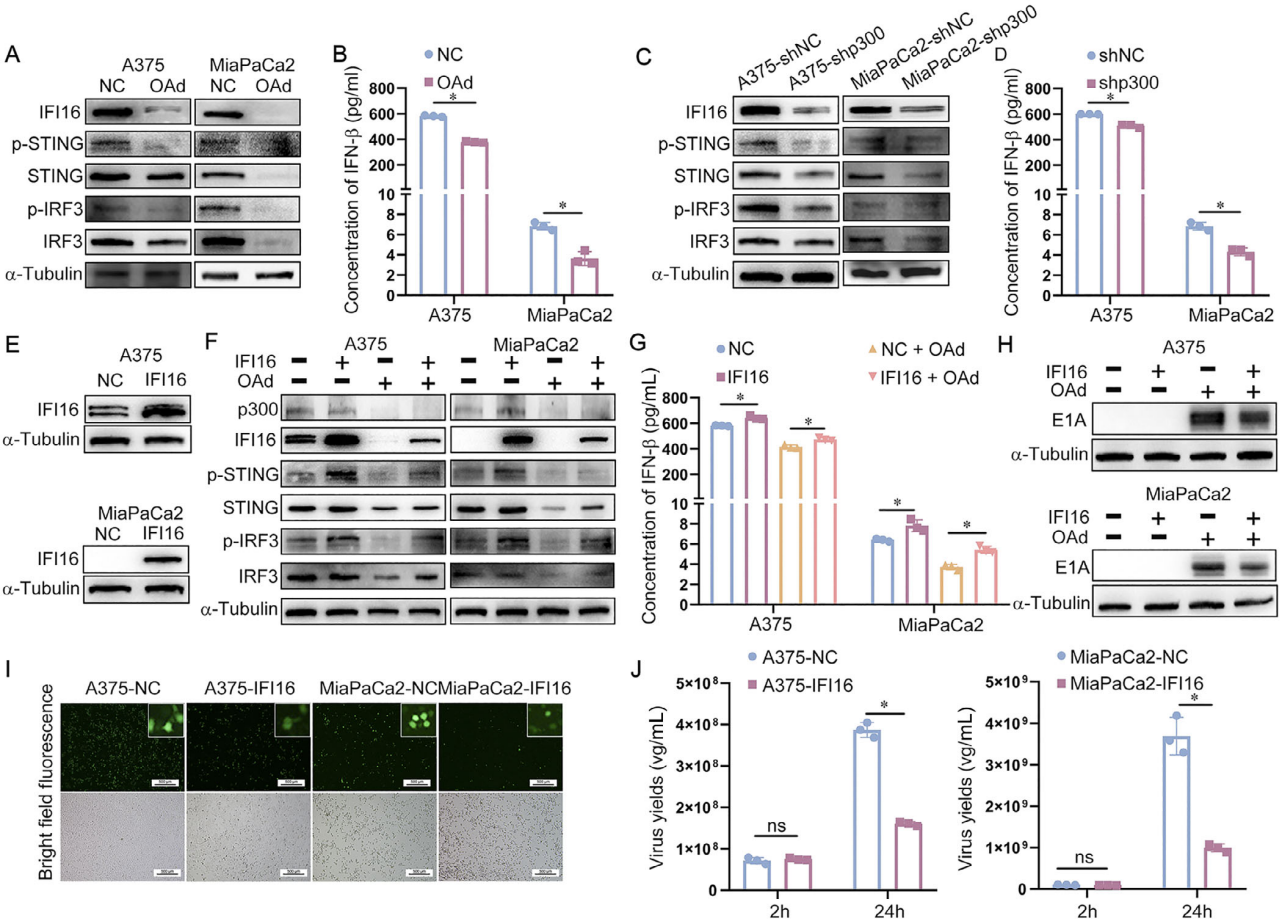
p300 regulates viral replication of oncolytic adenovirus by modulating the IFI16/STING/IRF3/IFN‐β signaling pathway. (A) Western immunoblotting was utilized to evaluate IFI16, p‐STING, STING, p‐IRF3, and IRF3 protein levels in A375 and MiaPaCa2 cells infected with OAd. (B) The expression of IFN‐β was detected using IFN‐β‐Elisa assay kit in A375 and MiaPaCa2 cells infected with OAd. (C) Western immunoblotting was used to detect IFI16, p‐STING, STING, p‐IRF3, and IRF3 protein levels in A375‐shp300 and MiaPaCa2‐shp300. (D) The expression of IFN‐β was detected using IFN‐β‐Elisa assay kit in A375‐shp300 and MiaPaCa2‐shp300. (E) Western immunoblotting was used to detect IFI16 in cells following its overexpression. (F) Western immunoblotting was used to detect p300, IFI16, p‐STING, STING, p‐IRF3, and IRF3 protein levels in A375‐NC, A375‐IFI16, MiaPaCa2‐NC, and MiaPaCa2‐IFI16 cells infected with OAd. (G) The expression of IFN‐β was detected using IFN‐β‐Elisa assay kit in A375‐NC, A375‐IFI16, MiaPaCa2‐NC, and MiaPaCa2‐IFI16 cells infected with OAd. (H) Western immunoblotting was used to detect E1A protein levels in A375‐NC, A375‐IFI16, MiaPaCa2‐NC, and MiaPaCa2‐IFI16 cells infected with OAd. (I) Following OAd‐EGFP for 24 h, cells were imaged via fluorescence microscopy. (J) qPCR was used to detect viral replication in A375‐NC, A375‐IFI16, MiaPaCa2‐NC, and MiaPaCa2‐IFI16 infected with OAd at 2 and 24 h post‐infection. Significant differences are denoted as *p < 0.05. α‐Tubulin served as the internal control.

The above studies have shown that p300 mainly regulates viral genome replication through IFI16. Thus, to study how p300 affects viral replication of OAd via IFI16, IFI16 and its downstream protein expression (STING, IRF3, and IFN‐β) were detected after A375 and MiaPaCa2 cell infection with OAd. The results showed that after infection with OAd in A375 and MiaPaCa2 cells, the expression of IFI16 decreased, and the phosphorylation of STING (p‐STING), phosphorylation of IRF3 (p‐IRF3), and IFN‐β expression decreased (Figures 5A,B). In addition, p300 knockdown could also reduce the expression of IFI16 and its downstream p‐STING, p‐IRF3, and IFN‐β (Figure 5C,D). Therefore, it suggests that E1A may promote adenovirus replication by inhibiting p300‐mediated IFI16/STING/IRF3/IFN‐β signaling pathway. To further validate the effect of p300/IFI16/STING/IRF3/IFN‐β signaling pathway on adenovirus replication, the overexpression of IFI16 cells (A375‐IFI16 and MiaPaCa2‐IFI16) was constructed (Figure 5E). Moreover, overexpression of IFI16 could attenuate the inhibitory effect of IFI16/STING/IRF3/IFN‐β signaling pathway caused by reduced p300 expression due to infection with OAd (Figure 5F,G). In addition, overexpression of IFI16 could reduce the expression of E1A (Figure 5H), weaken fluorescence intensity (Figure 5I), and inhibit viral replication (Figure 5J), indicating that overexpression of IFI16 can inhibit viral replication caused by E1A‐mediated reduction in p300 expression. Therefore, p300 inhibits viral replication of OAd via regulating the IFI16/STING/IRF3/IFN‐β signaling pathway.

### 2.6 Oncolytic Adenovirus With shRNA‐Targeting p300 Induces Efficient Viral Replication and Potent Antitumor Activity

**FIGURE 6:**
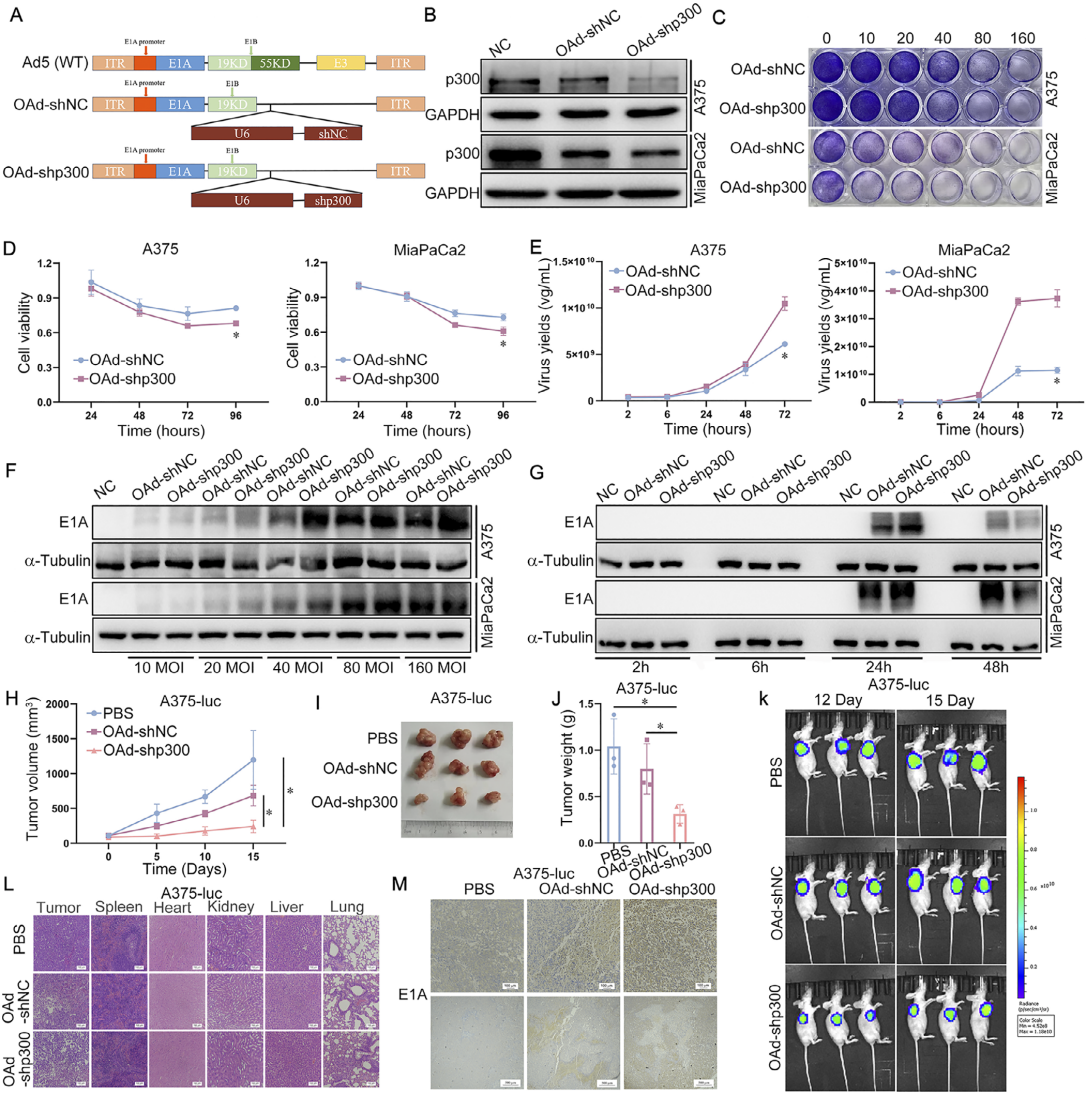
Oncolytic adenovirus with shRNA targeting p300 induces highly efficient viral replication and potent antitumor activity. (A) Schematic illustration of the structure of the recombinant oncolytic adenovirus. (B) E1A levels were assessed by Western immunoblotting. (C) Crystal violet staining was utilized to assess cytotoxicity of OAd‐shp300 toward A375 and MiaPaCa2 cells. (D) Following treatment with OAd‐shNC or OAd‐shp300 for the specified periods, MTT assays were used to assess A375 and MiaPaCa2 cell viability. (E) Following treatment with OAd‐shNC or OAd‐shp300 for the indicated periods, qPCR was used to detect viral replication in A375 and MiaPaCa2 cells. (F) Following treatment with OAd‐shNC or OAd‐shp300 at the indicated MOI, Western immunoblotting was utilized to evaluate E1A levels in A375 and MiaPaCa2 cells. (G) Following treatment with OAd‐shNC or OAd‐shp300 for the specified times, Western immunoblotting was used to evaluate E1A levels in A375 and MiaPaCa2 cells. (H) Tumor volumes were determined at 5‐day intervals in the different groups. (I) Tumor sizes in the different groups. (J) Tumor weights in the different groups. (K) Tumor growth measured by in vivo imaging. (L) Necrosis in tumors and normal tissues, as shown by HE staining. (M) IHC evaluation of E1A expression in tumor tissues. *p < 0.05. GAPDH and α‐tubulin served as the internal control.

Based on the mechanism of E1A promotion of OAd replication by degrading p300, OAd complexed with shRNA‐targeting p300 (OAd‐shp300) was constructed (Figure 6A). Relative to the OAd‐shNC group, p300 levels in the OAd‐shp300 group were decreased, indicating that OAd‐shp300 could effectively downregulate p300 (Figure 6B). Crystal violet (Figure 6C) and MTT (Figure 6D) assays further showed that relative to OAd‐shNC, cytotoxicity was greater in the OAd‐shp300 group. Moreover, relative to the OAd‐shNC group, replication of OAd was enhanced in the OAd‐shp300 group (Figure 6E) together with E1A expression relative to the OAd‐shNC group at different MOIs (Figure 6F) and times (Figure 6G), demonstrating the stronger viral replication capacity of OAd‐shp300. In vivo, OAd‐shp300 significantly suppressed tumor growth compared to OAd‐shNC (Figure 6H,I), while tumor weights were reduced in the OAd‐shp300 group compared to the OAd‐shNC group (Figure 6J). In vivo imaging also demonstrated that OAd‐shp300 significantly suppressed tumor growth compared to OAd‐shNC (Figure 6K). Relative to OAd‐shNC, OAd‐shp300 was more cytotoxic to tumor tissues, as shown by HE analyses, with minimal effect on normal tissues (Figure 6L). Notably, compared with OAd‐shNC, E1A expression was raised in the OAd‐shp300 group, indicating that OAd‐shp300 induced stronger replication in vivo (Figure 6M). These results indicated that OAd‐shp300 induced highly efficient viral replication and potent antitumor activity both in vitro and in vivo.

## 3 Discussion

Recently, OAd therapy has been identified as a novel cancer therapy after surgery, chemotherapy, and radiotherapy. It is an excellent vector for cancer gene therapy. For example, OAd encoding apolipoprotein A1 inhibits triple‐negative breast cancer metastasis in mice [19]. Ultralow‐dose binary oncolytic/helper‐dependent adenovirus promotes antitumor activity in preclinical and clinical studies [20]. OAd carrying SPAG9‐shRNA has been observed to enhance the efficacy of docetaxel in advanced prostate cancer [21]. In immunotherapy, OAd can turn cold tumors into hot tumors, allowing immune cells to infiltrate the tumor and exert antitumor effects [22]. OAd therapy can also improve the efficacy of anti‐PD‐1 monotherapy [23], while OAd vectors encoding human antibodies specific for PD‐1 can similarly achieve superior antitumor efficacy [24]. Additionally, OAd combined with chemotherapy [25, 26] or radiotherapy [27, 28] has better antitumor effects. In Phase III randomized clinical trial, intratumoral H101 injection combined with cisplatin plus 5‐fluorouracil (PF) regimen or adriamycin plus 5‐fluorouracil (AF) regimen showed a significant efficacy in patients with squamous cell cancer of head and neck or esophagus [29]. To sum up, it was observed that OAd plays a crucial role in combination therapy. However, previous clinical research studies have indicated that in OAd treatment alone, the therapeutic effect was not as potent [30]. Therefore, Efforts to study OAd biology are thus clearly warranted to better clarify the factors that can enhance the antitumor efficacy of this treatment strategy.

There are two major processes that influence the antitumor efficacy of OAd treatment. The first of these is the process of OAd entry into cells, which has been shown to be mediated by CAR receptors [31]. The second is the viral replication process. While E1A has been demonstrated to be required for adenoviral replication [10], the precise role that it plays in this process remains incompletely understood. The p300‐E1A‐RB1 complex can condense chromatin in a manner that is dependent on chromatin to repress host genes counterproductive to viral replication in a manner dependent on p300 lysine acetylase activity, HDAC activity, the p300 bromodomain, and RB K873/K874 and E1A K239 acetylation [16]. E1A conserved Region 3 is required to recruit p300 to the adenovirus E4 promoter during infection [15]. However, these studies did not use adenovirus to investigate its virus proliferation. Therefore, in this investigation, OAd was employed to evaluate the relationship between E1A, p300, and viral replication, providing comprehensive evidence for understanding adenovirus replication. The data revealed that after OAd infection, the expression of p300 decreased in the cells. However, after infection with non‐replicating adenovirus, the expression of p300 remains unchanged. Moreover, E1A overexpression in the cells reduced p300 expression, suggesting that E1A may promote p300 reduction by binding to p300. E1A has previously been demonstrated to be highly unstable within cells, such that it can readily undergo ubiquitination and consequent degradation [32]. In this study, E1A co‐precipitated with p300 in OAd‐infected cells, and the ubiquitination of p300 precipitated proteins was evident. The use of the proteasome inhibitor MG132 also effectively inhibited degradation of p300 in OAd‐infected cells while also hampering viral replication. Cells in which p300 had been knocked down presented with enhanced OAd replication and cytotoxicity. Together, these results provide support for a model wherein E1A can bind to p300 and facilitate its ubiquitination, ultimately leading to the proteasomal degradation of p300 and the corresponding enhancement of OAd replication. Notably, the expression of E1A decreases after reaching its peak, possible cytopathy leading to cell lysis and release of intracellular contents into the supernatant, resulting in a decrease in E1A. In addition, bioinformatics analysis revealed that p300 mainly affected IFI16, thereby affecting genes related to the negative regulation of viral genome replication. IFI16, as a restriction factor for viral replication of DNA viruses, inhibits the viral replication of DNA viruses [18, 33]. In this study, it was also found that E1A mediates p300 ubiquitination degradation, leading to a decrease in IFI16 expression, thereby inhibiting the IFI16/STING/IRF3/IFN‐β signaling pathway and ultimately enhancing viral replication of OAd.

Clinically, although the use of OAd alone in the treatment of tumors has achieved some results, it has not shown a good therapeutic effect in general [9, 30]. There are also no clear differences in the treatment outcomes of p53 mutant patients following OAd therapy as compared to p53 wild‐type patients [30]. This is consistent with reports suggesting that ONYX‐015 replication is independent of tumor cell p53 status [34]. At present, no clear biomarker indicative of OAd sensitivity has yet been established in cancer patients. However, in this study, p300 expression levels were associated with both OAd replication and cytotoxicity such that p300 knockdown cells or cells expressing low endogenous p300 levels (BxPC3, H1299) presented with greater viral replication and cell death as compared to cells in which p300 was endogenously expressed at high levels (MiaPaCa2, A375). Moreover, bioinformatics analysis showed that high p300 expression exhibits negative regulation of viral genome replication, consistent with the present findings. The overexpression of p300 can contribute to enhanced tumor progression [35], presenting a major barrier to effective OAd‐based tumor treatment. Efforts to explore whether peptide proteolysis‐targeting chimera (PROTAC) drugs or other agents can be used to target and downregulate p300 [36], potentially guiding the more appropriate OAd‐based treatment of individuals exhibiting p300 overexpression. In cases with lower levels of p300 expression, as observed in BxPC3 cells, OAd alone may be able to achieve good therapeutic effects. Based on these results, patients with low p300 expression should be prioritized for OAd treatment. In addition, the application of the OAd for treating tumors strongly expressing p300 requires a method for the effective and rapid reduction of p300 levels in the tumor to produce greater therapeutic effects. In this study, we designed OAd‐shp300 that could effectively reduce p300 expression, and both in vitro and in vivo experiments showed that relative to the control OAd‐shNC, OAd‐shp300 induced highly efficient viral replication and potent antitumor activity. Thus, reducing the expression of p300 may be an important factor in enhancing the antitumor properties of the OAd.

There are some limitations to these analyses. For one, these analyses only revealed a relationship between p300, E1A, and OAd replication. Additional research will be essential to clarify the precise mechanisms through which E1A controls the ubiquitination and degradation of p300 to facilitate viral replication. Further clinical studies will also be required to gain insight into the potential ability of p300 to function as a biomarker suitable for screening for cancer patients likely to be sensitive to OAd therapy.

## 4 Conclusion

**FIGURE 7:**
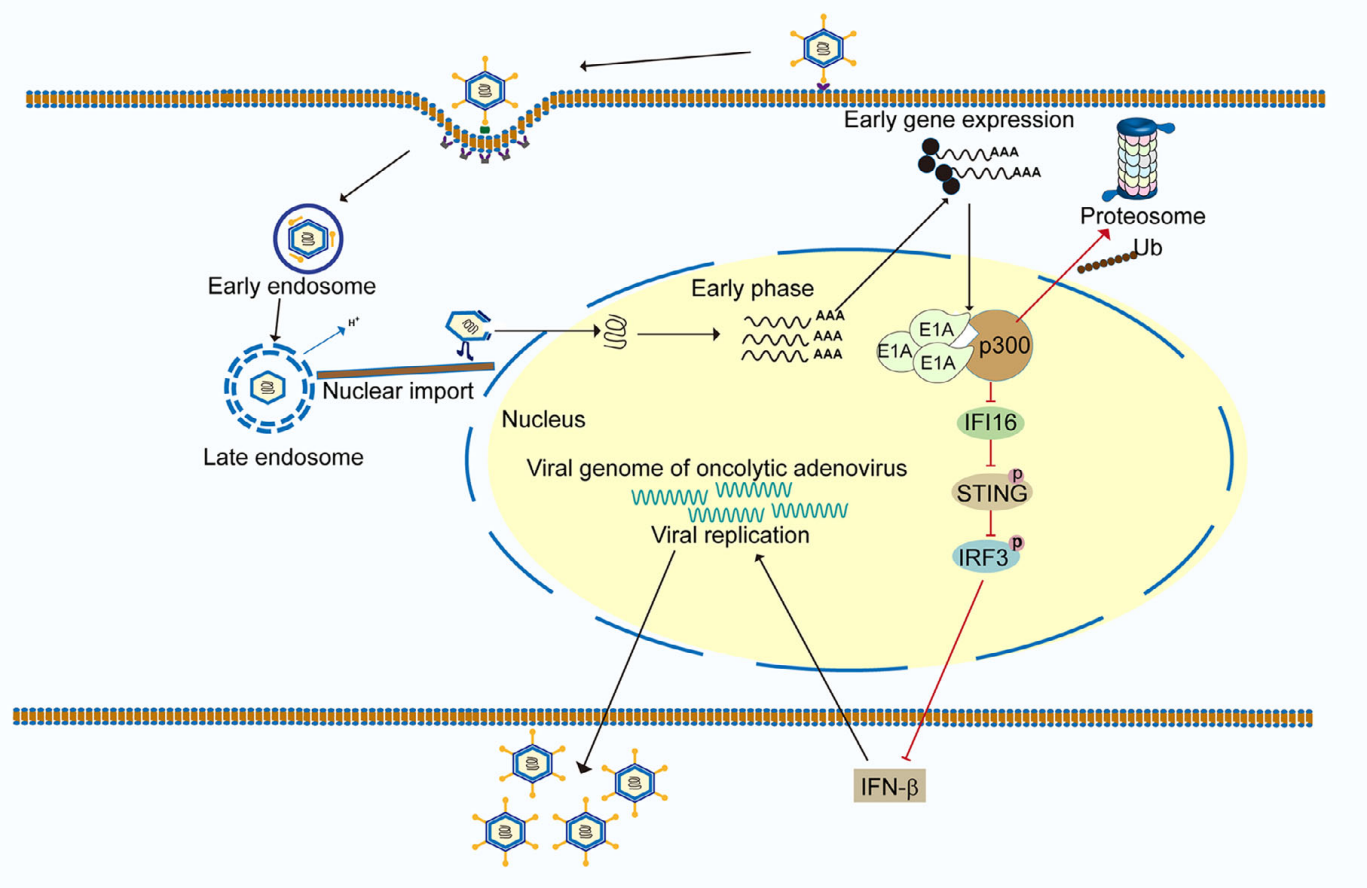
Schematic diagram of enhancer oncolytic adenovirus replication by regulating E1A/p300/IFI16/STING/IRF3/IFN‐β signaling pathway.

In summary, the present results revealed that OAd replication and cytotoxicity are enhanced in cells expressing low levels of p300. OAd infection can also degradate p300 through its E1A‐mediated ubiquitination, thereby inhibiting the IFI16/STING/IRF3/IFN‐β signaling pathway, ultimately supporting more robust viral replication and cell death (Figure 7). Together, these results thus provide a foundation that may enable screening for patients who are more likely to respond well to OAd treatment.

## 5 Materials and Methods

### 5.1 Cell and Culture

Human cancer cell lines (BxPC3, NCI‐H1299, MiaPaCa2, and A375) were provided by the Type Culture Collection of the Chinese Academy of Sciences (Shanghai, China) and cultured in DMEM (Gibco; Thermo Fisher Scientific, Inc., Waltham, MA, USA) augmented with fetal bovine serum (FBS; 10%; NEWZERUM; New Zealand) at 37°C and in 5% CO_2_.

### 5.2 Construction of OAd

The construction of OAd is as described in the previous method [37, 38]. In short, the E1 region, deleting E1B55kDa, was inserted into the pShuttle vector. Then, this vector was transformed into BJ5183 containing Adeasy‐1 for recombination. Finally, the genome of the obtained OAd was digested with PacI enzyme and then was transfected into HK293 cells to obtain the OAd.

### 5.3 Cell Viability Assay

The cells (5 × 10^3^/well) were cultured in 96‐well plates for 12 h and then treated with OAd (MOI = 10, 20, 40, 80, and 160). Cell viability was assessed at 24, 48, 72, and 96 h via methyl thiazolyl tetrazolium (MTT) assay. Briefly, the OAd‐treated cells were reacted with 20 µL MTT (5 mg/mL, Beyotime Biotechnology, China) for 4 h at 37°C. The MTT‐containing media was then aspirated, and dimethyl sulfoxide (150 µL) was added to each well. The optical density of each well was assessed using a microplate reader at 490 nm to calculate cell viability.

### 5.4 Western Blot Analysis

This analysis was performed by following the protocol described previously [39]. Briefly, after cell lysis, the proteins were quantified in the lysate via Pierce BCA protein assay kit, separated by SDS‐PAGE, transferred onto polyvinylidene difluoride membranes, and then probed with primary antibodies against E1A (Santa Cruz Biotechnology, USA), α‐tubulin (Proteintech, China), p300 (Abcam, UK), and GAPDH (Proteintech, China).

### 5.5 Virus Replication Assay

The OAd‐infected cells were collected at different time points to assess the viral titer. Vector copies were quantified with primer sets for OAd (F: 5′‐gtgacgtttttggtgtgcgc‐3′, R: 5′‐cgcgctatgagtaacacaaaattattcag‐3′). Based on the standard curve of original plasmid DNA, vector copies per microgram were assessed.

### 5.6 Immunofluorescence Assay

Immunofluorescence was carried out by following the method described previously [32]. Briefly, the cells were preserved for 30 min in 4% paraformaldehyde at ambient temperature, permeabilized with 0.5% Triton X‐100, occluded using 3% BSA in phosphate‐buffered saline (PBS), probed overnight with primary mouse α‐E1A and rabbit α‐p300 antibodies, and then treated with the secondary antibodies for 1 h at ambient temperature. The Zeiss LSM880 scanning confocal microscope was employed for cell analysis and imaging.

### 5.7 Immunoprecipitation Assay

The 24 h OAd‐infected cells were lysed with RIPA buffer. Then, the p300 antibody or IGg was used pull down the targeted protein overnight at 4°C. The p300 antibody‐dragged protein was detected using the Western blot with mouse anti‐E1A, mouse anti‐ubiquitination, and rabbit anti‐p300 antibodies.

### 5.8 Bioinformatics Analysis

The patient samples (GSE41372) and cell (CFPAC1 and Panc1) samples (GSE165949) of PDAC were obtained from NCBI's GEO database. The heatmap, GSEA, volcano plot, Venn diagram, and PPI were all analyzed using the R language.

### 5.9 Animal Experiments

BALB/c nude mice (4 weeks old, female) from Beijing Huafukang Bioscience (Beijing, China) were utilized to construct the xenograft model by subcutaneous administration of A375‐luc cells into the right flanks. When the tumors were 80–120 mm^3^, the animals were randomly assigned to three groups, with respective administration of PBS, OAd‐shNC (1 × 10^9^ pfu), and OAd‐shp300 (1 × 10^9^ pfu). Three doses (3 × 10^8^, 3 × 10^8^, and 4 × 10^8^ pfu) of OAd (1 × 10^9^ pfu) were given intratumorally once daily for three successive days. Tumor volumes were determined at 5‐day intervals with the formula *V* (mm^3^) = 1 / 2 × length × width^2^.

### 5.10 Histopathology and IHC

Randomly selected mice from each group were sacrificed after 15 days. Tissues, including the tumors and major organs (lungs, heart, kidneys, spleen, and liver), were collected, followed by fixation in 5% paraformaldehyde, dehydration in an ethanol gradient, paraffin‐embedding, and sectioning (5 µm). This was followed by haematoxylin and eosin staining for histopathological assessment. For IHC, after incubation with anti‐E1A antibodies and treatment with biotin and avidin‐peroxidase reagents (Vector Laboratories, Newark, CA, USA), the sections were counterstained with hematoxylin.

### 5.11 Statistical Analysis

All analyses were conducted using GraphPad Prism 8 software. The intergroup differences were evaluated via analysis of variance and Student's *t*‐test. A comparison of multiple groups was statistically evaluated via analysis of variance (ANOVA) and Tukey's post hoc test. All the statistical data are indicated as mean ± SD, and *p* < 0.05 was set as a statistically significant threshold.

## Author Contributions

B.X. and P.C. created study concept and design. B.X., Q.Y., S.H., J.H., Z.Q., Y.Z., and Y.Q. collected the data and analyzed and interpreted the results. B.X., Q.Y., and P.C. drafted the manuscript. All authors read and approved the final manuscript.

## Funding

This work was supported by the Innovative Drug Research and Development National Science and Technology Major Project (2025ZD1804204).

## Ethics Statement

Animal experiments were approved by the Experimental Animal Ethics Committee of West China Hospital of Sichuan University (No. 20240612004).

## Conflicts of Interest

The authors declare no conflicts of interest.

## Supporting information



Figure S1 DEGs analyzed by volcano plot.
Figure S2 A. Western immunoblotting was used to detect p300 protein levels in AsPAC1, BxPC3, Capan2, CFPAC1, MiaPaCa2, and Panc1 cells. α‐Tubulin served as the internal control. B. Following OAd treatment for the indicated periods, qPCR was used to detect viral replication in CFPAC1 and Panc1 cells.
Figure S3 p300 levels were upregulated in CFPAC1 cells compared to Panc1 cells.

## Data Availability

The data and constructs generated during this study are available from the corresponding author upon request.
