# Supplementary material for: Enhancing Oncolytic Adenovirus Replication by Early Region 1A Protein‐Mediated Degradation of E1A Binding Protein p300
Source: MedComm (2020). 2026 Mar 18;7(4):e70683. doi: 10.1002/mco2.70683 (PMC13042735; doi:10.1002/mco2.70683)
Supplement: Supplementary file 1 — Figure S1 DEGs analyzed by volcano plot. Figure S2 A. Western immunoblotting was used to detect p300 protein levels in AsPAC1, BxPC3, Capan2, CFPAC1, MiaPaCa2, and Panc1 cells. α‐Tubulin served as the internal control. B. Following OAd treatment for the indicated periods, qPCR was used to detect viral replication in CFPAC1 and Panc1 cells. Figure S3 p300 levels were upregulated in CFPAC1 cells compared to Panc1 cells. [file MCO2-7-e70683-s001.docx]

Enhancing oncolytic adenovirus replication by Early Region 1A Protein-mediated degradation of E1A binding protein p300


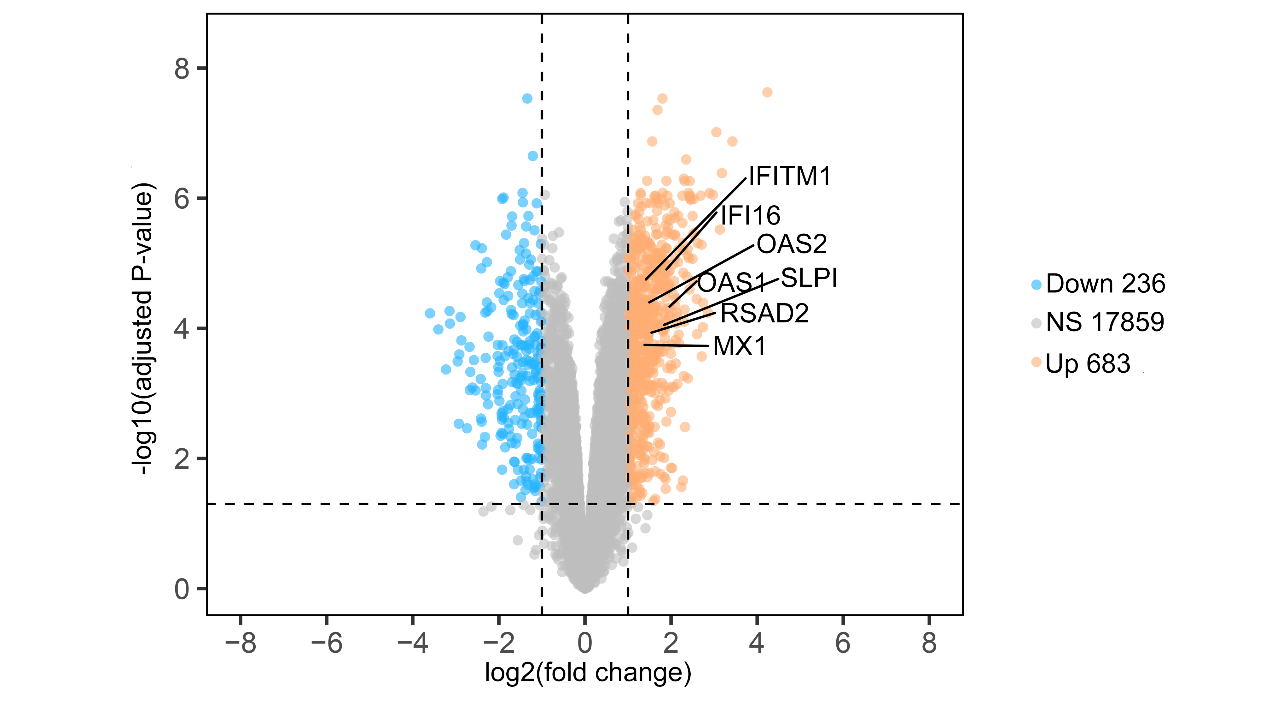


Figure S1. DEGs analyzed by volcano plot.


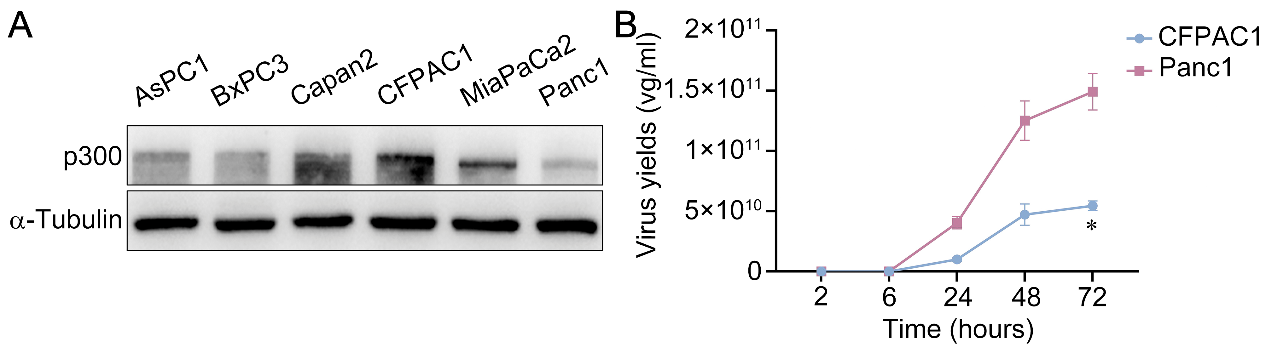


Figure S2. A. Western immunoblotting was used to detect p300 protein levels in AsPAC1, BxPC3, Capan2, CFPAC1, MiaPaCa2 and Panc1 cells. α-Tubulin served as the internal control. B. Following OAd treatment for the indicated periods, qPCR was used to detect viral replication in CFPAC1 and Panc1 cells.


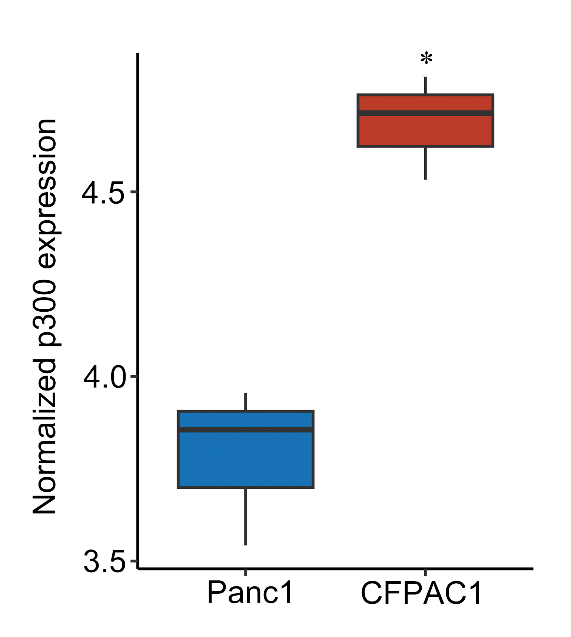


Figure S3. p300 levels were upregulated in CFPAC1 cells compared to Panc1 cells.
